# A DNA barcode reference library for CITES listed Malagasy *Dalbergia* species

**DOI:** 10.1002/ece3.9887

**Published:** 2023-03-15

**Authors:** Tahinarizaka Jenny Rakotonirina, Erika Viljoen, Antso Harimanantsoa Rakotonirina, Jean Michel Leong Pock Tsy, Tendro Radanielina

**Affiliations:** ^1^ Plant Molecular Biology Lab, Department of Plant Biology and Ecology University of Antananarivo Antananarivo Madagascar; ^2^ Inqaba Biotechnical Industries (Pty) Ltd Pretoria South Africa; ^3^ Département Forêt & Biodiversité Ambatobe FOFIFA Antananarivo Madagascar

**Keywords:** *Dalbergia*, DNA barcoding, illegal trade, *ITS*, *rbcL*

## Abstract

On Madagascar, the illegal and unsustainable exploitation and illegal international trade of *Dalbergia* (rosewood) precious woods remain a serious conservation problem. Members of this genus are at high risk of extinction as a consequence of logging, mining, and slash and burn agriculture. Morphological identification of these Malagasy species is difficult in the absence of flowers and fruits, especially in the case of cut trees, sawn wood, and finished product. In this study, we use molecular barcoding to identify the *Dalbergia* species with the intent to contribute to the control of their illegal trade. Thirty‐six *Dalbergia* samples representing 12 Malagasy species of which 11 have high commercial value, were collected to test the efficacy of a region of the plastid genome (*rbcL*) and a nuclear‐transcribed *ITS* for barcoding. These widely used markers, as well as DNA barcoding gaps, “best match” and “best close match” approaches, and the neighbor‐joining method were employed. All samples were amplified and sequenced using the two markers. Using a single locus, the “best match” and “best close match” approaches revealed that *ITS* has high discriminatory power within the tested Malagasy species. The combination of *rbcL* + *ITS* revealed 100% species discrimination. This study confirms that *ITS* alone and in combination with chloroplast barcode *rbcL* allow non‐ambiguous identification for the 12 species studied. The results contribute to the development of DNA barcoding as a useful tool to identify Malagasy *Dalbergia* and suggest that the approach developed should be expanded to all 56 potentially exploited species in reference to international CITES requirements and the sustainable management of valuable resources.

## Graphical abstract

The illegal and unsustainable exploitation and illegal international trade of Dalbergia (rosewood) precious woods remains a serious conservation problem. Thirty‐six Dalbergia samples representing 12 Dalbergia species from Madagascar were collected to test the efficacy of a region plastid genome (rbcL) and a nuclear transcribed spacer ITS used as barcode. This study shows that ITS alone and in combination with chloroplast barcode rbcL+ITS allow 12 Dalbergia Malagasy species identifications and encourages for the development of DNA barcoding as a useful tool to identify Malagasy Dalbergia species to the CITES requirements.

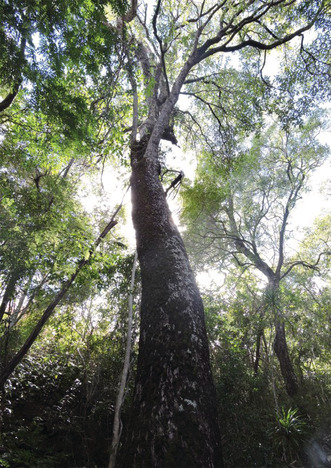

## 1 INTRODUCTION

The wood of genus *Dalbergia* (Fabaceae), commonly known as rosewood and palisander, is highly appreciated because of its physical (hardness) and chemical properties (non‐rotting), which making it high of commercial value (Bhagwat et al., 2015). Wood from Malagasy *Dalbergia* is mostly used for furniture and wood timber (Andrianoelina et al., 2009). The genus on Madagascar comprises 83 species, 55 of which are published, and 56 of these 83 species are large enough trees to be potential sources of commercially valuable wood (Madagascar Catalogue, 2022a, 2022b). The *Dalbergia* species are distributed across all bioclimatic regions of Madagascar, including dry deciduous forests, humid forests, subhumid forests, and succulent woodlands (Madagascar Catalogue, 2022a, 2022b).

At a global scale, the rate of deforestation and illegal logging in major timber‐producing regions is a critical threat to biodiversity and forest ecosystems (Jiao et al., 2018). Since 2009, the illegal exploitation of these precious woods has continued to increase (Waeber & Wilmé, 2013). They are at high risk of extinction because of significant levels of logging, mining, and other human activities such as swidden (slash and burn) agriculture and charcoal production (Barrett et al., 2010; Styger et al., 2007). In 2013, Malagasy members of the genus were included in the Convention on International Trade in Endangered Species (CITES) Appendix II, and in 2016 because of the significant number of illegal exports, international trade of these species was suspended. The Convention on International Trade in Endangered Species has recommended that Madagascar makes progress on both the scientific and governance aspects on Decision 18.96 (CITES, 2016). The adulteration of forest products increases illegal trafficking and creates a serious problem for species management and conservation (Wiedenhoeft et al., 2019). More specifically, confusion in the identification of traded *Dalbergia* wood as compared to similar non‐threatened species occurs (Hartvig et al., 2015), a situation that is accentuated by the lack of identification tools and a comprehensive reference library.

The genus *Dalbergia*, belonging to the family Fabaceae, subfamily Papilionoideae, is a taxonomically complex genus because in the absence of flowers and fruits it is difficult to distinguish species from one other based on morphological characteristics (Hassold et al., 2016), especially in the case of cut trees, sawn wood, and finished products. To obtain reliable identification, we have explored the use of molecular techniques. A global barcoding initiative to standardize molecular identifications using DNA protocols and regions has been agreed upon at the international level: The Consortium for the DNA Barcode of Life (CBOL) (CBOL Plant Working Group et al., 2009; Hebert et al., 2003). DNA barcoding has already been tested on Malagasy *Dalbergia* and on species from other countries, and was shown to be effective for their discrimination and identification using *ITS* (Hartvig et al., 2015; Phong et al., 2014; Yu et al., 2016) and *rbcL* and *matK* (Bhagwat et al., 2015; Hassold et al., 2016) gene regions. According by Hassold et al. (2016), DNA barcoding is an effective method for discriminating Malagasy *Dalbergia* from members of the genus occurring in other countries.

This study aims to (i) develop and test DNA barcoding as a tool for identifying Malagasy *Dalbergia* species using the chloroplastic region *rbcL* and a nuclear region *ITS*; and (ii) establish a reference database for 12 Malagasy *Dalbergia* species using barcodes to provide a basis for reliable and accurate identification in order to control their illegal exploitation and trade.

## 2 MATERIALS AND METHODS

### 2.1 Plant material

**FIGURE 1:**
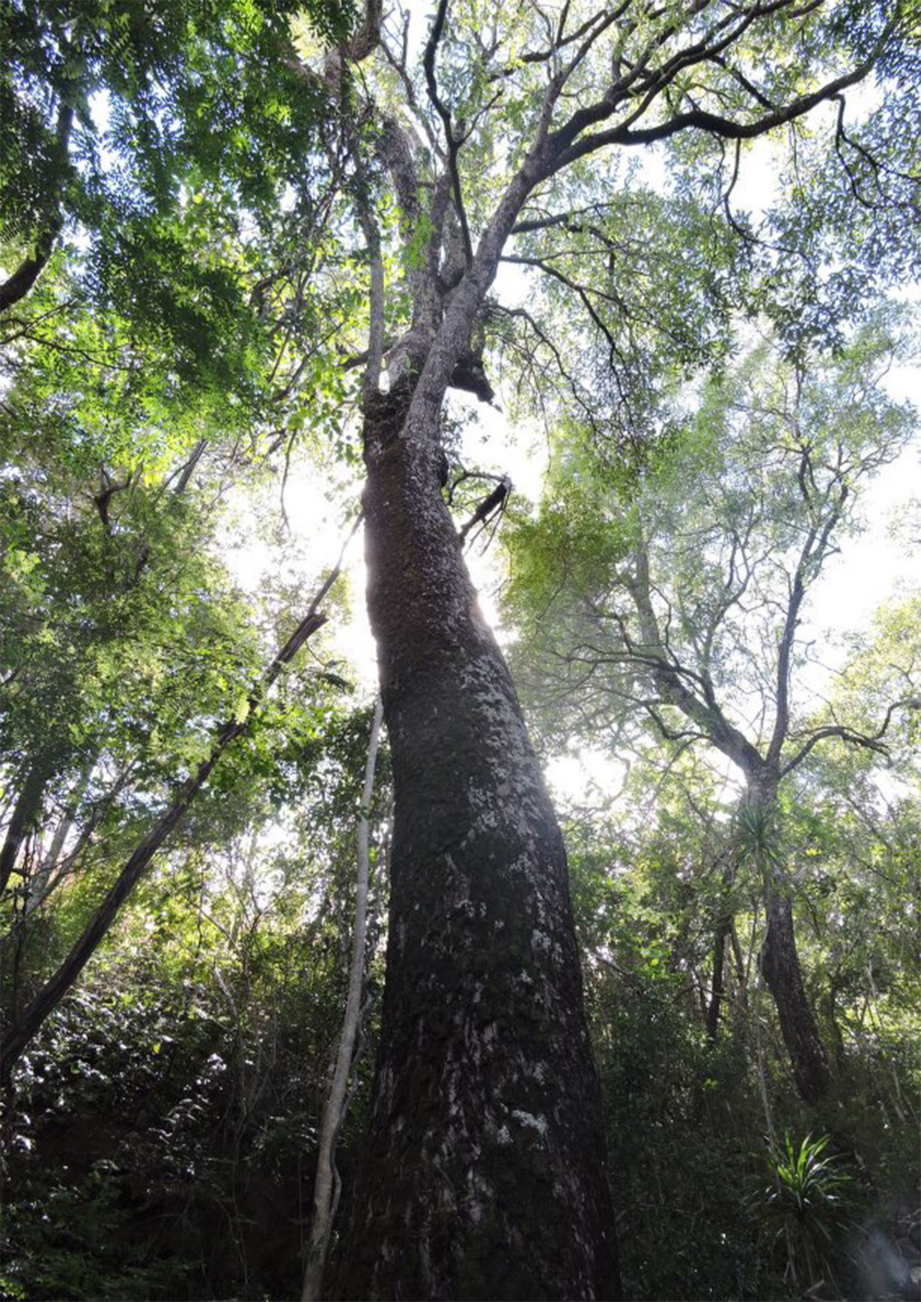
*Dalbergia tricolor* photographed by Richard RANDRIANAIVO

**FIGURE 2:**
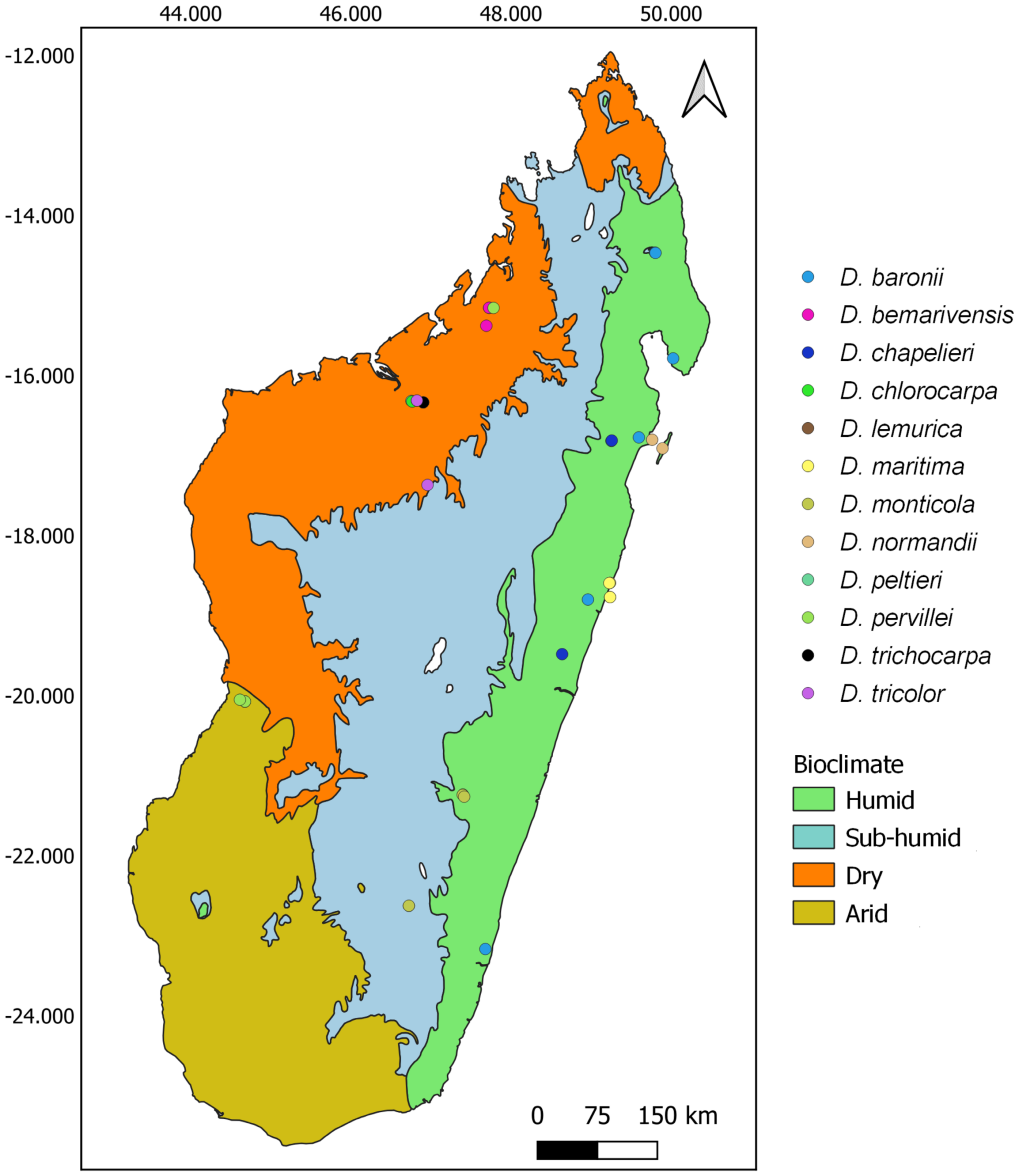
Collection sites for *Dalbergia* species on Madagascar

Since 2019, an EU‐funded project has enabled the collection of material of *Dalbergia* throughout Madagascar; more than 2000 samples have been collected from all over the island (Figure 1). From these samples, 12 species (Figure 2) were selected for this study and represented by 36 samples from young and healthy leaf material (Appendix S1) that were immediately dried and stored in silica gel until DNA extraction. Each collected sample, represented by a specimen deposited in the Parc Botanique et Zoologique de Tsimbazaza (TAN) herbarium, was morphologically identified by the collaborating plant taxonomists at the Missouri Botanical Garden based on their leaves, fruits, and flowers.

### 2.2 Molecular methods

#### 2.2.1 DNA extraction, amplification, and sequencing

Genomic DNA was isolated from 20 mg of silica‐dried *Dalbergia* leaf samples using the Plant/ Seed DNA Isolation Kit (Zymo Research) following the manufacturer's protocol. The quality and concentration of DNA were evaluated using a nanodrop spectrophotometer (Thermo Fischer Scientific).

As previously indicated, two barcoding gene regions were investigated, chloroplast *rbcL* and nuclear *ITS* (Dong et al., 2012; Hartvig et al., 2015). The *rbcL* and *ITS* primers are listed in Table 1. Primer oligonucleotides were synthesized at Inqaba Biotechnical Industries (Pty) Ltd.

**TABLE 1:** Barcoding primers used in this study

Barcode gene	PCR primers	Primer sequence 5′‐3′	References
*rbc*L	RbcLa‐F	ATGTCACCACAAACAGAGACTAAAGC	Levin et al. (2003)
	RbcLa‐R	GTAAAATCAAGTCCACCRCG	Kress & Erickson (2007)
*ITS*	ITS2‐S2F	ATGCGATACTTGGTGTGAAT	Chen et al. (2010)
	ITS4	TCCTCCGCTTATTGATATGC	White et al. (1990)

PCR amplification was performed using the Mastercycler® Nexus gradient (Eppendorf). The PCR reactions were conducted in a final volume of 20 μL containing New England Biolabs (NEB) Onetaq 2× Mastermix (10 μL), forward primer (1 μL, 10 μM), reverse primer (1 μL, 10 μM), and nuclease‐free water (7 μL), DNA (1 μL, 15–30 ng/μL). The amplification conditions consisted of an initial denaturation at 94°C for 5 min, following 35 cycles of denaturation at 94°C for 30 s, annealing gradient at 50–55°C for 1 min, extension at 68°C for 1 min, and final extension at 68°C for 10 min. After amplification, the DNA integrity was evaluated by 1% agarose gel electrophoresis stained with 0.5 μg/mL ethidium bromide.

Gel images were obtained using an imaging system (Cleaver Scientific). Amplified PCR products were enzymatically purified using ExoSAP‐IT™ (NEB). Fragments were prepared for sequencing using the BrilliantDye™ Terminator Cycle Sequencing Kit V3.1 (Nijmegen, Netherlands). The labeled products were purified with the ZR‐96 DNA Sequencing Clean‐up Kit (Zymo Research). The purified products were injected into the Applied Biosystems ABI 3500XL Genetic Analyzer (Thermo Fischer Scientific) with a 50 cm array, using POP7™ Polymer (Thermo Fischer Scientific). Samples were sequenced with the forward primer only. Wet lab experiments were performed at Inqaba Biotechnical Industries (Pty.) Ltd. Sequence chromatogram viewing/analysis was performed using FinchTV analysis software.

#### 2.2.2 Data analysis

The DNA sequences for the *ITS* and *rbcL* regions of all samples were edited individually and manually using BioEdit software (Hall, 1999). After editing, the FASTA sequences were aligned using the Clustal‐W algorithm with default parameters in Molecular Evolutionary Genetics Analysis‐X (MEGA‐X) (Kumar et al., 2018). The alignments were manually adjusted as needed with BioEdit Software (Hall, 1999).

Intraspecific and interspecific distances were calculated using the MEGA‐X software for the two individual gene regions and their combination. The sequence barcoding approach is very useful for discriminating sister species, where the amount of variation must be sufficient and not affecting their correct assignation based on intraspecific variation (Laiou et al., 2013). The barcoding gap is calculated by the difference between the minimum interspecific distance and the maximum intraspecific distance, the former value must be higher than the latter (Zhang et al., 2015). If barcoding gap is detected, the species can be regarded as well differentiated (Meier et al., 2008).

To evaluate species discrimination success, two methods were used, Best Match and Best Close Match in TaxonDNA software (Meier et al., 2006) and a neighbor‐joining (NJ) tree‐based approach (Saitou & Nei, 1987) in MEGA software; these were applied to the two single barcodes (*rbc*L and *ITS*) as well as the combination (*ITS + rbcL*) under the P‐distance model.

For the tree‐based method, unrooted NJ trees were constructed in MEGA‐X with pairwise deletion and the Kimura 2‐parameter model according to published sources on species discrimination (Hartvig et al., 2015; He et al., 2019; Kumar et al., 2018; Yu et al., 2017) and nodal support was determined through bootstrap analysis with 1000 replicates (Saitou & Nei, 1987). For the 36 specimens used, if all the conspecific individuals clustered in a single clade, this was considered a correct species discrimination.

TaxonDNA/SpeciesIdentifer 1.8 (Meier et al., 2006) was used for DNA barcoding analysis. This software calculates intraspecific and interspecific genetic distances, matching sequences, and clustering sequences based on pairwise distances. For analyzing identification rates of DNA barcodes, the criteria “best match”, “best close match”, and “all species barcodes” were employed (Meier et al., 2006). For the TaxonDNA analysis, we used the “best match” and the “best close match” functions in the software to test the species‐level discrimination rates under the Kimura 2‐parameter corrected distance model for each barcode singly and all possible combinations of barcodes. The “best close match” method required a threshold value, which was calculated for each barcode from the pairwise summary. All the results above the threshold were treated as “no match”.

## 3 RESULTS

### 3.1 Variation of barcoding markers

Genomic DNA extraction was successful for all 36 *Dalbergia* samples. PCR amplification was performed on the chloroplastic marker *rbcL* and one nuclear marker *ITS*. All PCR products were sequenced in a forward direction for *ITS* and *rbcL*. A total of 72 sequences were obtained from the 12 *Dalbergia* species. After trimming and alignment, the sequence lengths observed in all the analyzed samples included 428 bp for *rbcL* and 334 bp for *ITS* (Table 2). The combined alignment had a total length of 762 bp, of which 670 bp were constant sites, 87 bp variable sites, and 72 bp parsimoniously informative sites. Mean intraspecific and interspecific distances were, respectively, 0.0034469 and 0.0702978 for *ITS* and 0.0006851 and 0.0067735 for *rbcL* (Table 2).

**TABLE 2:** Comparisons variability of the two DNA markers

DNA region	*ITS*	*rbc*L
Percentage PCR success	100%	100%
Aligned sequence length (bp)	334	428
No. sampled species (individuals)	12 (36)	12(36)
Variable site	75	12
Conserved site	254	416
Parsimony informative site	63	9
Mean interspecific distance	0.0702978	0.0067735
Mean intraspecific distance	0.0034469	0.0006851

### 3.2 Genetic distance and barcoding gap

**FIGURE 3:**
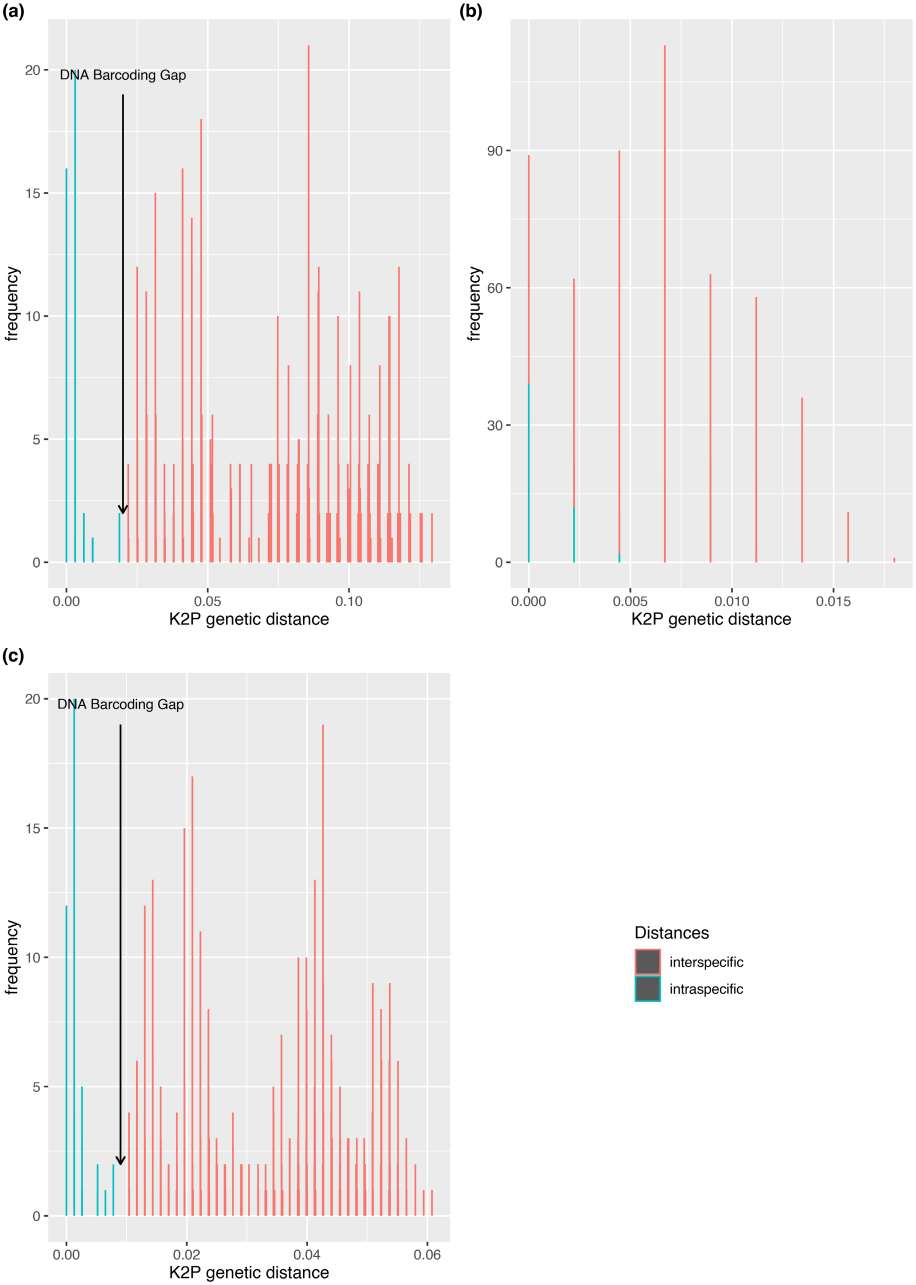
Evaluation of DNA barcoding gap. Graph of minimum interspecific and maximum intraspecific distances highlighting the overlapping divergence. X axes relate to Kimura 2‐parameter (K2P) distances arranged in intervals, and the Y axes correspond to the loci. (a) *ITS*, (b) *rbcL*, (c) *ITS + rbcL*

Intraspecific and interspecific distances were calculated by the MEGA X software for two regions. The range of maximum intraspecific distance is 0.0044580–0.0187593 and the minimum interspecific distance is 0–0.218746 for each locus and combined (Table 4). The barcoding gap represents the absence of overlapping regions between interspecific and intraspecific distances. The values of the maximum intraspecific and minimum interspecific sequence divergence of the barcoding loci and their combination are shown in Figure 3. The results indicate that both *ITS* (0.004740687) individually and in combination with the chloroplast marker *ITS + rbcL* (0.00440638) show a barcoding gap (Figure 3). These results for *ITS* and in combination with the chloroplast marker (*ITS* + *rbcL*) can be used as a barcode to discriminate among the examined Malagasy *Dalbergia* species. The barcoding gap was not observed for *rbcL* alone (Figure 3), indicating that there is insufficient interspecific variation in this marker compared to the level of intraspecific. However, average interspecific divergence was significantly higher than the corresponding intraspecific divergence for each of the loci, which was confirmed by the TaxonDNA analysis (Table 3).

**TABLE 3:** Identification success based on the ‘best match’ and ‘best close match’ function of the program TaxonDNA

DNA region	Best match			Best close match		
	Successfully identified (%)	Ambiguous (%)	Misidentified (%)	Successfully identified (%)	Ambiguous (%)	Misidentified (%)
*RbcL*	38.88	58.33	2.77	38.88	58.33	2.77
*ITS*	100	0	0	100	0	0
*rbcL + ITS*	100	0	0	100	0	0

### 3.3 Application for species discrimination

TaxonDNA and NJ trees were used to discriminate between the 12 *Dalbergia* species studied. Considering the “best match” and “best close match” analysis with the TaxonDNA software, the highest discriminatory power between the species using the two markers is *ITS* (100%), whereas that for *rbcL* is lower (38.88%; Table 3). The combination of *ITS* + *rbcL* revealed 100% species discrimination (Table 3). This study showed that among the candidate loci, *ITS* and the combination *ITS* + *rbcL* provided the best discriminating power between the tested Malagasy *Dalbergia* species.

**FIGURE 4:**
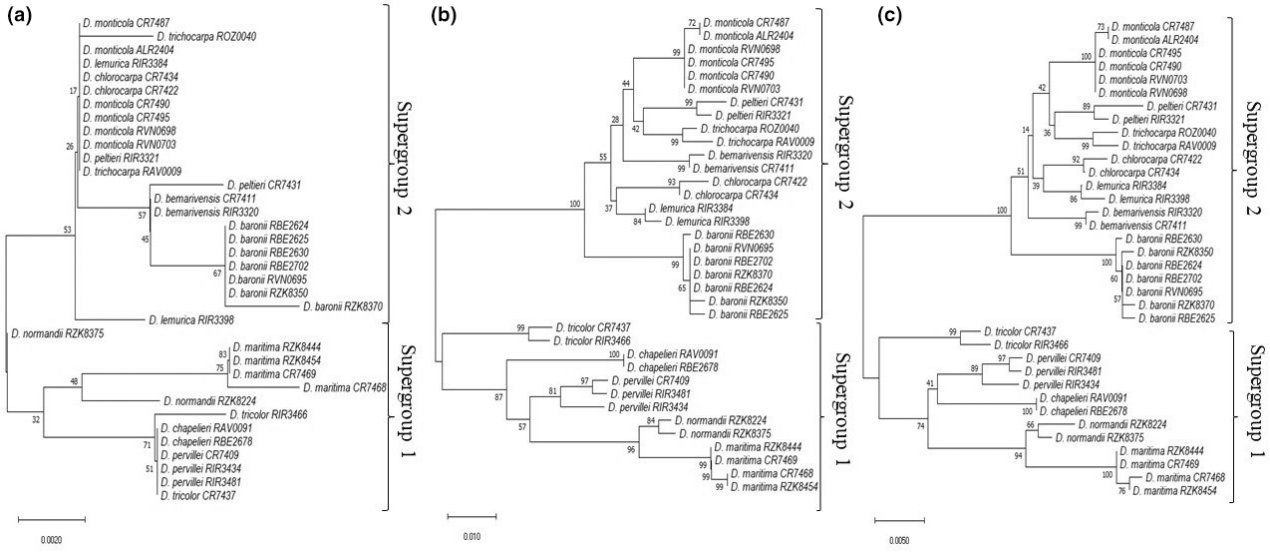
Neighbor‐joining tree based on K2P distance model. (a) *rbcL*; (b) *ITS*; (c) *rbcL + ITS*. Bootstrap values (>50%) are shown above the relevant branches

On the basis of the methods used herein, the molecular grouping of individuals identified as the same species based on herbarium specimens and as belonging to the same clade, which indicates a correspondence between morphological and molecular identification. On the basis of the NJ trees, the tested *Dalbergia* species are divided into two groups; one compromising members supergroup 1, as defined by Hassold et al. (2016) including *D. chapelieri*, *D. maritima*, *D. normandii*, *D. pervillei*, and *D. tricolor*, and the supergroup 2, *D. baronii*, *D. bemarivensis*, *D. chlorocarpa*, *D. lemurica*, *D. monticola*, *D. peltieri*, and *D*. *trichocarpa* (Figure 4). The use of a single marker, *ITS* in this case, provides a clear discrimination of *Dalbergia* species (Figure 4). The combination of *ITS* with *rbcL* does not provide any greater level of discrimination (Figure 4).

## 4 DISCUSSION

International trade of *Dalbergia* species from Madagascar has been officially suspended since 2016 because of the high level of illegal exportation (CITES, 2016). The CITES action plan (Decision 18.96) was adopted for *Dalbergia spp*. that Madagascar shall continue the production of identification materials for timber and timber products for species of this genus (CITES, 2018). Our results indicate that DNA barcoding provides a clear manner to identify the 12 tested Malagasy *Dalbergia* species, which include 11 of the principal commercially valuable species except *D. peltieri*.

Recent taxonomic revisions have shown that there are 56 endemic *Dalbergia* species of potential trade value on Madagascar (Madagascar Catalogue, 2022a, 2022b). The results of Vatanparast et al. (2013) on the phylogeny of *Dalbergia* indicate that the Malagasy species are divided into three clades, which also include the various African species. This aspect was confirmed by Hassold et al. (2016) and Crameri (2020) who concluded that, the two identified Malagasy clades diverge from African, American, and Asian *Dalbergia*, and the island was the subject of at least two independent colonization events. Our results contribute to clarifying the identification of 12 species on the 56 endemic Malagasy *Dalbergia* species having important trade value.

DNA barcoding involves the use of short regions of DNA to identify biological materials to species by classifying individuals of known taxa by comparing their sequence data with those from a reference library (Saarela et al., 2013). An ideal DNA barcode should have the following characteristics: amplification capacity with universal primers, high amplification and sequencing efficiency, and enough genetic variation to distinguish sequences at the species level, but also conservative enough not to confound intraspecific variation (CBOL Plant Working Group, 2009).

*ITS* was initially proposed as a DNA barcode marker for plants because of its high sequence divergence (Kress et al., 2005). Studies on vascular plants have shown that it has high species discrimination and the combination of *ITS* with plastid DNA markers was able to differentiate between species groups (China Plant BOL Group et al., 2011). DNA barcoding allowed the identification of *Dalbergia* species using *ITS* from several areas (Hartvig et al., 2015; He et al., 2019; Phong et al., 2014; Yu et al., 2016). In the subtribe Cassiinae (Fabaceae), *ITS* was suggested as the first option for DNA barcoding (Mishra et al., 2016). In the current study, the Best Match and Best Close Match analysis with the TaxonDNA software revealed that the discriminatory power of the two markers at the species level for 12 different *Dalbergia* taxa is 100% for *ITS* and 38.88% for *rbcL*. The *rbcL* region has been proposed as a barcode to discriminate among species because of its wide use, ease of amplification and alignment, and the existence of considerable online sequence data of numerous taxa (Hollingsworth et al., 2011; Newmaster et al., 2006). Our results are similar to those reported in work done on tropical tree species in India, where *rbcL* was found to have a high PCR and sequencing success rate, but a low species discrimination power of 43.6% (Tripathi et al., 2013). For studies on other species of *Dalbergia*, the low discrimination power of *rbcL* has been observed by other researchers (Hassold et al., 2016; Liu et al., 2017).

With a 100% identification success rate of the 12 *Dalbergia* species studied here, *ITS* can be used to identify Malagasy *Dalbergia* to the species level. Similar results were found of nine *Dalbergia* species from Africa, Asia, and Central America, which revealed a 97.6% (Yu et al., 2017) and 99.2% (He et al., 2019) identification success rate with *ITS* alone. For other genera of plants, *ITS* also has a high discriminatory power of 86.7% (Yang et al., 2012).

The current study also showed that, the combination of *ITS* + *rbcL* sequence data likewise provided 100% species identification. Similar results were found in other *Dalbergia* species using a combination of chloroplast and nuclear markers, *ITS* + matK+*rbcL* (Hartvig et al., 2015) and *ITS* + *trnV + trnM1* + *trnH‐psbA* and *ITS2* + *trnL* (Yu et al., 2017). Previous research has indicated that using a single locus, *ITS* allows the highest discrimination of *Dalbergia* species although *ITS* in combination with *rbcL* and *matK* provides the best discrimination (Hartvig et al., 2015). The higher discriminatory power of the nuclear DNA region *ITS* over plastid barcodes has also been observed in *Pterocarpus*, with 85.1% correct species determination (Jiao et al., 2018).

Based on *rbcL* data from the 12 Malagasy *Dalbergia* species examined herein, the range of intraspecific distance was from 0 to 0.018759 and that of interspecific distance from 0 to 0.12937691 (Table 4). We did not identify a barcoding gap for *rbcL* (Figure 3). However, the mean interspecific divergence was significantly higher than the corresponding mean intraspecific divergence for each of the loci, which was confirmed by the TaxonDNA analysis (Table 3). These results are similar to those presented in the study of Hassold et al. (2016) using the chloroplast marker *rbcL*. Research conducted on different *Dalbergia* species from Asia and America showed a barcoding gap using *ITS* by itself and in combination with the other markers (Bhagwat et al., 2015; Hartvig et al., 2015).

**TABLE 4:** Genetic distance generated using Kimura 2‐parameter model analysis for the candidate barcode loci and its combination

Single and combined barcode	Intraspecific distance			Interspecific distance		
	Minimum	Maximum	Mean	Minimum	Maximum	Mean
*ITS*	0	0.0187593	0.0034469	0.0218746	0.1293769	0.0702978
*RbcL*	0	0.0044580	0.0006851	0	0.0179936	0.0067735
*ITS + rbcL*	0	0.0077936	0.0018310	0.0103965	0.0607610	0.0324550

Phylogenetic analysis can reveal whether species are monophyletic species, as well as providing important insights into species discriminations (Nithaniyal et al., 2014). Our phylogenetic trees can also be used for differentiating species with *ITS* and in combination with *rbcL*. Our analyses confirm that the species of Malagasy *Dalbergia* are divided into two distinct groups (Crameri, 2020; Hassold et al., 2016; Vatanparast et al., 2013). The recent studies performed on Malagasy members of this genus concur with our phylogenetic analyses in that supergroup 1 includes *D. tricolor*, *D. chapelieri*, *D. pervillei*, *D. maritima*, and *D. normandii* and supergroup 2 comprises *D. monticola*, *D. peltieri*, *D. trichocarpa*, *D. bemarivensis*, *D. chlorocarpa*, *D. lemurica*, and *D. baronii* (Crameri, 2020). Our results are consistent with and complementary to previous studies by Crameri (2020) and Hassold et al. (2016) regarding the phylogenetic position of the 12 species analyzed herein.

Referring to the results of the NJ phylogenetic trees with respect to the identification of *Dalbergia* from other parts of the world, the use of a single *ITS* locus showed a high identification rate (75%) and was even better when combined with other markers (100%) (He et al., 2019), which is similar to our findings. Taking into account the node support values, *ITS* alone and in combination with *rbcL* shows the same results for the discrimination of supergroup 1 species. On the other hand, for supergroup 2, *ITS* alone gives better results with high node‐support values in discriminating the closely related species of *D. maritima* and *D. normandii*. Elsewhere in the world, *ITS* failed to discriminate between Asian species *D. tonkinensis* and *D. odorifera* (Yu et al., 2016). One of the important roles of a sequence database is to help determine the identity of unidentified material (Meier et al., 2006). To test the performance of molecular identification, it is necessary to perform a blind test in which an unknown sample is analyzed and sequenced and then compared with the available sequences of previously identified samples.

Madagascar is still facing a difficult task to control exportation of its own precious hardwoods and to control illegal exploitation and illegal international trade. A national decree (Decree no 2016‐801) prohibits the cutting, exploitation, and exportation of Malagasy *Dalbergia* species which are listed in Appendix II of CITES. The mixing of more than one species, either combined in a finished product or in raw form in a shipping container, represents a considerable challenge for the Malagasy authorities and for customs and regulatory authorities in countries through which material transits or into which it is being imported in the application of CITES. In any case, there is a need for an accurate method to identify material before exportation. In addition, challenges arise when certifying the legality of these wood products because of difficulties in differentiating very similar species. The CITES legislation and global trade monitoring require precise species‐level identification and geographic traceability of wood (Dormontt et al., 2015). DNA barcoding can play a clear role in the identification of *Dalbergia* species, as well as other precious wood groups at a worldwide scale (Jiao et al., 2018). The availability of molecular sequences from positively identified reference samples can be used as an identification tool for customs officers at ports to ensure the effective identification of exported rosewood to determine its legality. The current study prioritized 12 *Dalbergia* species that are widely used in the large commercial trade, as an initial step toward establishing reference sequences for all 56 exploited species in Madagascar using the DNA barcoding method. Improved methods in the identification of *Dalbergia* species are an essential step in advancing the needs of CITES authorities, stakeholder, customs border and users.

Our results show that the use of a *ITS* marker by itself or in combination with *rbcL* provides efficient identification of the 12 Malagasy *Dalbergia* species examined, 11 of which are of high commercial value. It will now be necessary to extend this test study to all the large tree species of *Dalbergia* in Madagascar with the available samples. Precise identification of Malagasy *Dalbergia* by DNA barcoding may be an important factor in increasing the trade value of legally exploited and exported timber species, promoting innovative silvicultural approaches for regeneration and conservation, and supporting new, profitable industries within Madagascar. The Malagasy government has officially indicated its commitment to promoting the sustainable use of the country's precious woods. The utilization of this technique will make an important contribution to efforts aimed and ensuring the conservation of these species. The use of DNA barcoding as an identification tool provides reliable identifications accurately. In order to ensure this essential capacity, Madagascar needs appropriate in‐country facilities and expertise, along with adequate financial resources to operate and maintain them.

## AUTHOR CONTRIBUTIONS

**Tahinarizaka Jenny RAKOTONIRINA:** Conceptualization (lead); data curation (lead); formal analysis (lead); investigation (equal); methodology (lead); software (lead); writing – original draft (lead); writing – review and editing (lead). **Erika Viljoen:** Resources (equal); supervision (equal); validation (equal); writing – original draft (supporting); writing – review and editing (equal). **Harimanantsoa Antso RAKOTONIRINA:** Resources (supporting); software (supporting); validation (equal). **Jean Michel LEONG POCK TSY:** Resources (equal); software (supporting); validation (equal); visualization (equal). **Tendro RADANIELINA:** Conceptualization (equal); funding acquisition (equal); methodology (equal); resources (equal); supervision (equal); validation (equal); writing – review and editing (equal).

## CONFLICT OF INTEREST STATEMENT

The authors declare no conflicts of interest.

## Supporting information


Appendix S1.

## Data Availability

DNA sequences: Genbank accessions ON226895‐ON226930; ON212229‐ON212264 and BOLD accessions DALB001‐22‐ DALB036‐22
