# Supplementary material for: A DNA barcode reference library for CITES listed Malagasy Dalbergia species
Source: Ecol Evol. 2023 Mar 15;13(3):e9887. doi: 10.1002/ece3.9887 (PMC10015365; doi:10.1002/ece3.9887)
Supplement: Supplementary file 1 — Appendix S1. [file ECE3-13-e9887-s001.docx]

**Appendix S1:** Sample information with Genbank accession numbers. List of taxon names, Voucher information, locality and Genbank accession numbers for the 36 Malagasy *Dalbergia* samples in the present study. Column “Voucher” gives the acronym of the public herbarium where vouchers are deposited.

| **Specimen voucher** | **Species identification** | **Region** | **Location** | **IUCN**  **status of assessments** | **Genbank accession numbers** | |
| --- | --- | --- | --- | --- | --- | --- |
|  |  |  |  |  | ***ITS*** | ***RBCL*** |
| RBE2624 | *Dalbergia baronii* | SAVA | Marojejy | **Vulnerable** | ON226895 | ON212229 |
| RBE2625 | *Dalbergia baronii* | SAVA | Marojejy | **Vulnerable** | ON226896 | ON212230 |
| RBE2630 | *Dalbergia baronii* | Analanjirofo | Ambanizana | **Vulnerable** | ON226897 | ON212231 |
| RBE2702 | *Dalbergia baronii* | Analanjirofo | Ambatovaky | **Vulnerable** | ON226898 | ON212232 |
| RVN0695 | *Dalbergia baronii* | Atsimo-Antsinanana | Agnalazaha | **Vulnerable** | ON226899 | ON212233 |
| RZK8350 | *Dalbergia baronii* | Analanjirofo | Ambatovaky | **Vulnerable** | ON226900 | ON212234 |
| RZK8370 | *Dalbergia baronii* | Antsinanana | Sahafina | **Vulnerable** | ON226901 | ON212235 |
| CR7411 | *Dalbergia bemarivensis* | Sofia | Anjiamangirana | Critically endangered | ON226902 | ON212236 |
| RIR3320 | *Dalbergia bemarivensis* | Sofia | Anjiamangirana | Critically endangered | ON226903 | ON212237 |
| RAV0091 | *Dalbergia chapelieri* | Antsinanana | Antanambao Manampotsy | Near threatened | ON226904 | ON212238 |
| RBE2678 | *Dalbergia chapelieri* | Analanjirofo | Ambatovaky | Near threatened | ON226905 | ON212239 |
| CR7422 | *Dalbergia chlorocarpa* | Boeny | Ankarafantsika | Critically endangered | ON226906 | ON212240 |
| CR7434 | *Dalbergia chlorocarpa* | Boeny | Ankarafantsika | Critically endangered | ON226907 | ON212241 |
| RIR3384 | *Dalbergia lemurica* | Menabe | Kirindy | Vulnerable | ON226908 | ON212242 |
| RIR3398 | *Dalbergia lemurica* | Menabe | Kirindy | Vulnerable | ON226909 | ON212243 |
| CR7468 | *Dalbergia maritima* | Antsinanana | Andranampy | Endangered | ON226910 | ON212244 |
| CR7469 | *Dalbergia maritima* | Antsinanana | Vohibola | Endangered | ON226911 | ON212245 |
| RZK8444 | *Dalbergia maritima* | Antsinanana | Andranampy | Endangered | ON226912 | ON212246 |
| RZK8454 | *Dalbergia maritima* | Antsinanana | Vohibola | Endangered | ON226913 | ON212247 |
| ALR2404 | *Dalbergia monticola* | Haute Matsiatra | Sahamalaotra | Vulnerable | ON226914 | ON212248 |
| CR7487 | *Dalbergia monticola* | Ihorombe | Vohiboro | Vulnerable | ON226915 | ON212249 |
| CR7490 | *Dalbergia monticola* | Haute Matsiatra | Sahamalaotra | Vulnerable | ON226916 | ON212250 |
| CR7495 | *Dalbergia monticola* | Ihorombe | Vohiboro | Vulnerable | ON226917 | ON212251 |
| RVN0698 | *Dalbergia monticola* | Haute Matsiatra | Sahamalaotra | Vulnerable | ON226918 | ON212252 |
| RVN0703 | *Dalbergia monticola* | Vatovavy Fitovinany | Ranomafana | Vulnerable | ON226919 | ON212253 |
| RZK8224 | *Dalbergia normandii* | Analanjirofo | Ambodimanga | Endangered | ON226920 | ON212254 |
| RZK8375 | *Dalbergia normandii* | Analanjirofo | Sainte Marie | Endangered | ON226921 | ON212255 |
| CR7431 | *Dalbergia peltieri* | Boeny | Ankarafantsika | **Vulnerable** | ON226922 | ON212256 |
| RIR3321 | *Dalbergia peltieri* | Sofia | Anjiamangirana | **Vulnerable** | ON226923 | ON212257 |
| CR7409 | *Dalbergia pervillei* | Sofia | Anjiamangirana | **Vulnerable** | ON226924 | ON212258 |
| RIR3434 | *Dalbergia pervillei* | Betsiboka | Ambalanjanakomby | **Vulnerable** | ON226925 | ON212259 |
| RIR3481 | *Dalbergia pervillei* | Menabe | Kirindy | **Vulnerable** | ON226926 | ON212260 |
| ROZ0009 | *Dalbergia trichocarpa* | Boeny | Ankarafantsika | **Least concern** | ON226927 | ON212261 |
| ROZ0040 | *Dalbergia trichocarpa* | Boeny | Ampondrabe | **Least concern** | ON226928 | ON212262 |
| CR7437 | *Dalbergia tricolor* | Boeny | Ankarafantsika | **Vulnerable** | ON226929 | ON212263 |
| RIR3466 | *Dalbergia tricolor* | Betsiboka | Andoharina | **Vulnerable** | ON226930 | ON212264 |
